# Evidence for Seroprevalence in Human Localized Cutaneous Leishmaniasis Caused by* Leishmania donovani* in Sri Lanka

**DOI:** 10.1155/2018/9320367

**Published:** 2018-01-17

**Authors:** Yamuna Deepani Siriwardana, Bhagya Deepachandi, Samantha Ranasinghe, Preethi Soysa, Nadira Karunaweera

**Affiliations:** ^1^Department of Parasitology, Faculty of Medicine, University of Colombo, Colombo, Sri Lanka; ^2^Ministry of Health, Colombo, Sri Lanka; ^3^Department of Biochemistry and Molecular Biology, Faculty of Medicine, University of Colombo, Colombo, Sri Lanka

## Abstract

Visceral leishmaniasis (VL) is considered as a major health threat in the Indian subcontinent.* Leishmania donovani*, a usually visceralizing species, causes cutaneous leishmaniasis (CL) in Sri Lanka. However, visceralizing potential of the local* L. donovani* is not yet fully understood. This project studied the seroprevalence of local CL by using an in-house ELISA. An IgG-based ELISA using crude* Leishmania* antigen (Ag) was developed and validated. A total of 50 laboratory confirmed cases of locally acquired CL were examined using the newly developed ELISA. According to the optimized ELISA, seroprevalence of anti-*Leishmania* IgG antibodies in the study group was 34.0% (*n* = 17/50). Majority of seropositive individuals were males (*n* = 13/17), representing 76%. Nearly half of the seropositive individuals were young adults (20–40 years, *n* = 9/17, 53%). Higher proportions of single lesions, large lesions, and nodular lesions were associated with a seroconversion. A proportion of local* L. donovani *infections leading to CL have the ability to raise an antibody response in the host. This may indicate early systemic involvement as one possibility. Study of a large number of patients with adequate follow-up would be useful.

## 1. Introduction

Leishmaniasis is caused by several species of the genus* Leishmania.* Clinical outcome takes one of the three main forms, that is, cutaneous (CL), visceral (VL) and mucocutaneous (MCL) leishmaniasis, mainly depending on the infecting species [1]. CL and MCL mainly affect the skin and mucosal tissues, respectively. First-line adaptive cellular resistance exerted by the host in response to* Leishmania* infection is predominantly Th1-associated in CL and Th2-associated in VL. Significantly elevated humoral response patterns in response to various antigens have been mainly described for visceral leishmaniasis [2, 3]. Diagnostic tools that detect this serological response are often used in the laboratory identification of VL.* L. donovani* complex specific rK39 dipstick assay is a frequently used serological tool for VL detection [4–6].

Apart from its traditionally known clinical outcome leading to VL,* L. donovani* is occasionally known to cause CL [7–10]. However, many studies have shown that CL detection rate of rK39 assay is low [11, 12]. ELISAs have been developed for the detection of CL with varying results [50.0% to 98.0% positivity] [13, 14]. Literature on CL caused by* L. donovani* is scarce.

Human leishmaniasis caused by* L. donovani* is considered as an established disease in Sri Lanka [15–17]. Local* L. donovani* parasite was found to be genetically different from known* L. donovani* strains in other endemic settings [17]. CL remains the main clinical entity in Sri Lanka [18–20] with only few reported cases of VL and MCL [21, 22]. Ability of the local CL parasites to raise an antibody response has not been studied. In a previous attempt to elicit such a response using rK39 immunochromatographic test on 24 cases of CL, none of them turned positive [10]. This indicated a poor or absent humoral response or, on the contrary, possible undetected antigenic differences of the local parasite variant. This project was designed to examine the serological response using an IgG-based in-house ELISA. Current study describes the first attempt to develop an in-house ELISA and its use to determine the prevalence of anti-*Leishmania* IgG antibodies in CL acquired in Sri Lanka.

## 2. Materials and Methods

### 2.1. Sample Collection

A total of 90 patients who presented to the routine leishmaniasis clinic at the Department of Parasitology, Faculty of Medicine, University of Colombo, with single or multiple skin lesions suggestive of cutaneous leishmaniasis were recruited after obtainment of informed written consent. They were clinically evaluated and data were gathered using a standard pretested questionnaire. Lesion aspirations (LA), slit-skin scrapings (SSS), and/or punch biopsies (PB) were collected from clinically suggestive skin lesions. Diagnosis of CL was established by light microscopic examination of LAs and scrapings. PCR was carried out on DNA extracted from punch biopsies of all microscopy negative patients [23, 24].

### 2.2. Preparation of Crude Protein Lysate

*Leishmania* parasites were grown in M 199 culture medium according to the previously established protocols [24]. Cell pellets were prepared by centrifugation of parasites in late log phase at 3000 rpm for 10 minutes. Cells of a single culture isolate were used throughout the study to minimize errors. Crude protein lysate was obtained from the cell pellet by freeze-thaw method. Cell pellet was dissolved in sterile 1x PBS. Rapid freeze-thaw cycles were performed using liquid nitrogen. Pellet was dipped in liquid nitrogen until the freezing temperature was achieved. The pellet was then taken out and kept at 37°C for 1 minute approximately until being completely dissolved. Procedure was repeated thrice to lyse proteins. Lysate was sonicated for 20 seconds followed by centrifugation and the supernatant was used for subsequent procedures. Protein estimation was carried out according to the modified Lowry procedure [25].

### 2.3. Sera for Positive and Negative Controls

A volume of 2 ml of intravenous blood was collected to a plain tube from a local patient with locally acquired and parasitologically confirmed VL (bone marrow examination by microscopy on direct smear and* in vitro* cultures), 20 apparently healthy individuals (HC) residing in a nonleishmanial area (Colombo), and ten patients with other skin diseases which clinically mimic CL (NCL) in the local setting (psoriasis, 2, leprosy, 3, eczema, 3, and allergic dermatitis, 2). Blood was allowed to clot at room temperature and centrifuged at 3000 rpm for 10 minutes. Serum was separated and stored at −20°C for further use. Sera from the VL patient and 20 HCs were examined by rK39 assay and confirmed positive in case of VL and negative in all HCs. HC samples were used as normal human serum for ELISA optimization. Sera of patients having other skin diseases (NCL) were tested for determination of cross reactions of the optimized ELISA.

### 2.4. ELISA Protocol/Methodology

Checker board titration method was used. Different quantities of antigen (i.e., 0.5 *μ*g, 1.0 *μ*g, and 1.5 *μ*g) produced similar absorbance values. Two HRP concentrations were tested (1/8000 and 1/16000). Patient sera were tested at 1/2000, 1/4000, and 1/8000 dilutions. Best concentrations of antigen, HRP, and patient sera were determined.

### 2.5. ELISA Protocol

A 96-well ELISA plate (Nunc, USA) was coated with 1.0 *μ*g/well/100 *μ*l of Ag in 0.05 M carbonate buffer at pH 9.6. The plate was incubated overnight at 4°C. Following overnight incubation, the plate was washed off 3 times with 0.1% Tween-20 in 1x PBS (PBST). Washed plate was coated with 200 *μ*l of 5% skimmed milk in 1x PBS (blocking buffer) and incubated at room temperature for 2 hours. Patient sera (100 *μ*l/well, 1 : 8000 dilution in blocking buffer) were added and incubated overnight at +4°C, followed by three washes with PBST. Following overnight incubation, wells were washed thrice with 1x PBST as previously. Plate was coated with 100 *μ*l of HRP-Goat Anti-Human IgG conjugate (Invitrogen, 1/16000 dilution) and incubated at 37°C for 30 minutes. The plate was then washed with 1x PBST for 5 times at 5-minute intervals between each washing. Substrate TMB (Invitrogen) was added (100 *μ*l/well) and incubated for 30 minutes at room temperature in dark conditions for color development. Reactions were stopped after adding the stop solution (1 N sulphuric acid/100 *μ*l). Absorbance values were read at 450 nm using ELISA reader (Thermo Electron Corporation Multiskan EX).

### 2.6. Optimization and Validation of ELISA Results

Optimization was followed by validation of the assay. Triplicate determinations for each test sample were carried out. Data were accepted if there were closer values to the second decimal place. Positive control sample was tested in the same microtiter plate and a standard curve was obtained. ELISA values for each sample and linearity of the assay were determined using the standard curve. Linearity was determined by analysis of six replicates of five different concentrations (concentration ranged from about 80% to 120% of the expected concentration range) of positive control. In determining repeatability of the assay, positive and negative controls were tested in each ELISA plate within the study period in order to normalize the data without any plate-to-plate or day-to-day variations. The lowest concentration that could be reliably measured by the test was determined using the standard ELISA parameters, limit of blank (LoB) and limit of detection (LoD) calculated using the standard equations; LOB = *M*_Blank_ + 1.645(SD_Blank_); LOD = LOB + 1.645(SD_low concentration of analyte_).

**Figure 1:**
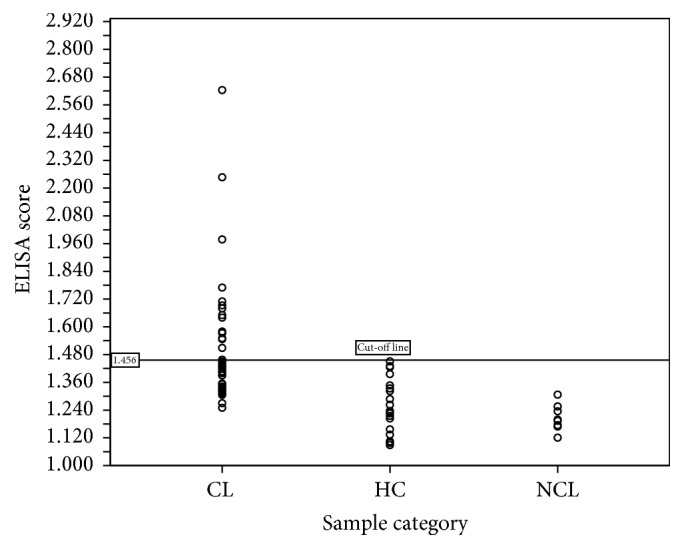
Distribution of enzyme-linked immunosorbent assay (ELISA) response of cutaneous leishmaniasis (CL), healthy control (HC), and non-CL (NCL) isolates. Positive control had a mean absorbance value of 2.400, while control samples, HC, and NCL had ELISA scores less than 1.456.

Mean ELISA value for the assay was decided based on the values obtained for 20 nonimmune serum samples. Two standard deviations (2SD) were added to the mean OD value of healthy controls (*M*) to obtain the final cut-off value for the assay. Baseline absorbance value for the assay was determined as 1.456 (*M* + 2SD) based on the values obtained for 10 nonimmune serum samples. Positive control had a mean absorbance value of 2.4. All the NCL samples (*n* = 10) had ELISA scores less than 1.47 (Figure 1). Therefore the cross reactions were considered as minimum in the optimized assay. Within the tested one-week period, the repeatability of the assay was 100%.

The accuracy of the method was also determined relative to the gold standard LM, using standard calculation methods (accuracy = true positives and true negatives/total number of samples). Also, receiver operating curve (ROC) analysis was used to determine sensitivity and specificity of the assay. Percentage of study sample of CL giving OD values in ELISA above the cut-off value was taken as the seroprevalence.

### 2.7. Data Analysis and Statistical Analysis

SPSS statistical software was used to analyze clinical data and laboratory results. Seropositivity rate of the study group was determined. Seropositivity data were compared with the clinical features by crosstabulation.

*Ethical Aspects*. Ethical clearance for the study was obtained from Faculty of Medicine, University of Colombo. Data and sample collection were carried out following obtainment of informed consent from patients.

## 3. Results

### 3.1. Study Sample and Laboratory Confirmation

A total of 90 patients with clinically suspected CL were recruited. A majority of the study population consisted of males (*n* = 73/90, 81.1%) and aged between 12 and 73 (mean: 36.6) years. They represented different regions including Northern (*n* = 28/90, 31.1%) and Southern (*n* = 38/90, 42.2%) provinces. Mean lesion duration was 8.5 months. Majority of patients had single lesions (*n* = 63/90, 70.0%). Multiple lesion types were observed (papules (*n* = 7/90, 7.8%), nodules (*n* = 26/90, 28.9%), ulcerating nodules (*n* = 18/90, 20.0%), plaques (*n* = 14/90, 15.6%), ulcers (*n* = 14/90, 15.6%), and other types (*n* = 11/90, 12.2%)). The diagnosis for leishmaniasis was confirmed in *n* = 50/90 (55.6%). From the confirmed CL group, *n* = 17/50 (34.0%), seropositivity was observed at 1.456 cut-off level.

#### 3.1.1. ELISA Results

**Figure 2:**
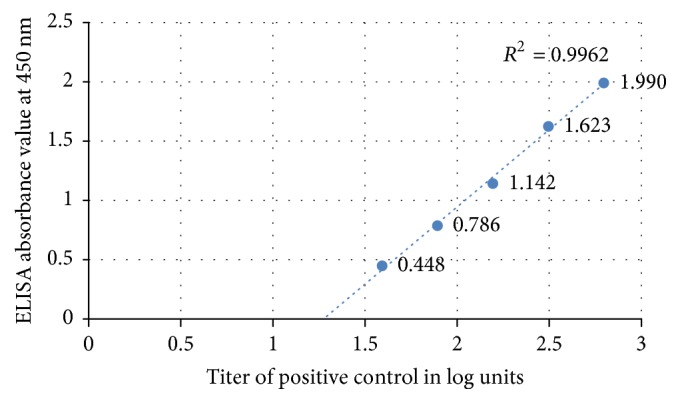
Standard curve for positive control. The linearity of the assay was shown by coefficient of determination, *R*^2^ (=0.9962) calculated using the standard curve.

**Figure 3:**
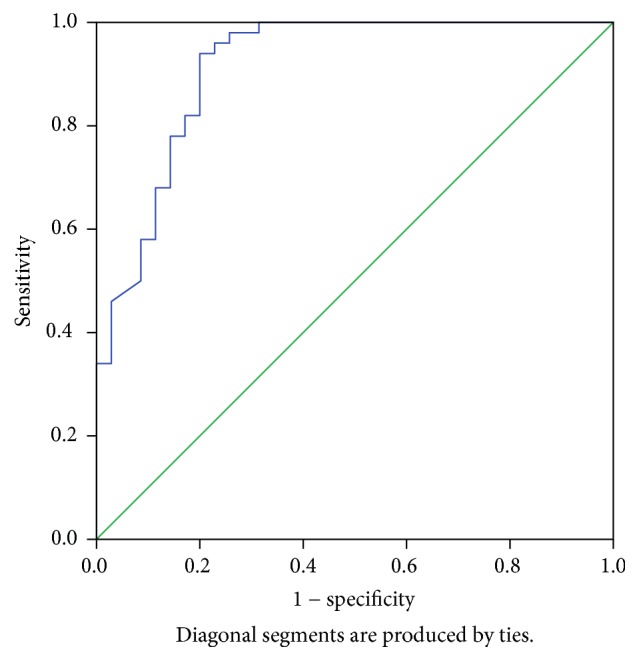
Receiver operating curve (ROC curve) analysis. Test sensitivity and specificity were 94.0% and 80.0%, respectively, at 1.308 cut-off level.

*R*-squared value (*R*^2^) or coefficient of determination calculated using the standard curve was 0.9962 (Figure 2). The linearity should yield a correlation coefficient > 0.9 according to the standard guidelines. The LoB and LoD of the assay were calculated as 0.005 and 0.034, respectively. Also the test was 52.0% accurate in diagnosis as compared to the gold standard, LM (Table 1). According to the ROC curve analysis, test sensitivity and specificity were found to be 94.0% and 80.0%, respectively, at 1.308 cut-off level (Figure 3). Area under the curve for ROC analysis was 0.915 at 95% confidence interval.

Among the parasitologically confirmed cases of CL, ELISA values were found to be over 1.456 in seventeen patients (17/50, 34.0%). It was determined that there is a 34.0% seroconversion rate with regard to IgG response in* L. donovani* skin lesions included in this study.

#### 3.1.2. Clinical and Epidemiological Correlations

Nearly 40.0% of patients from Northern province and 36.0% of those from Southern province were seropositive. Nearly one-third (6/20) of military personnel and 40% (11/28) of civilians were seropositive (Table 2). Majority of seropositive individuals had single lesions, while 13/50 individuals had multiple lesions. Higher proportion of very small (≤2 cm, 35.8%) lesions produced a serological response as compared to the seropositive proportions of larger (>2 cm) lesions. Among patients, one-third of males (*n* = 13/40, 32.5%) and 40.0% (*n* = 4/10) of females were found to be seropositive. All the lesion developmental stages were associated with seroconversion (Table 2).

## 4. Discussion

There is 34% seroprevalence of anti-*Leishmania* antibodies among the 50 cases of locally acquired and laboratory confirmed patients with CL. Causative organism of CL in Sri Lanka was identified as* L. donovani* earlier [17]. Studies have demonstrated ranging figures for different CL endemic settings [4–6]. However, observed seroconversion in a minority of CL patients in the study indicates a potential for visceralization at least in a proportion of CL cases which may be reflected through systemic immune response and transient immune response for circulating antigens in otherwise localized CL or concurrent asymptomatic VL together with CL. This warrants in-depth studies with adequate case numbers and follow-ups, since members of* L. donovani* complex except for* L. infantum* usually result in VL, while cutaneous lesions attributed to the same species complex are only occasional. Visceralization of usually dermotropic* Leishmania *species is also a known phenomenon [26]. Recent reported locally transmitted VL and MCL already provide supportive evidence for possible visceralization of at least a minority of* L. donovani* infections in Sri Lanka. However, an immune response to parasitemia or circulating antigens is more likely, since long-term follow-up studies on local CL have failed to demonstrate evidence for visceralization in humans or in mouse model [27]. Progressive skin infection remains the main mode of clinical presentation of local parasite so far [10, 15, 18]. Therefore, the ability of a few* Leishmania* parasites that escape into the blood stream to raise an antibody response while major pathology is confined to the skin is the most likely possibility [13, 14]. Subsequent visceralization of CL caused by* L. donovani* in other settings has not been reported yet. Degree of humoral response in CL is variable [13]. Therefore, current findings in the local setting warrant further investigation.

Clinical and sociodemographic data of seropositive individuals indicated that both genders, all age groups, and multiple lesion types are associated with varying degrees of serological response in cases occurring in different areas in the island.

However, in the selected study group, high seroconversion rates (SCR) were seen in cases reported from both North and South. Most of the local* L. donovani* infections still originate from these two areas. Serological response in nonimmune populations acquiring infection from these areas also needs to be studied using a more representative sample. The place of acquisition of infection of soldiers from Northern Sri Lanka was considered as their working environment (where they spent most of the days of a given month favouring this possibility). Nearly a half of them showed a serological response in the current study. Further analysis revealed that soldiers represented a single age group (21–30 years), probably reflecting the age distribution within their occupation, while civilians were of a wider age range (data not shown). Previous studies have reported higher involvement of age group of 21–40 years with CL as compared to other age groups [19]. There may be variation in antibody response patterns in* Leishmania* strains or patient populations prevalent in different areas.

Elderly individuals showed a low serological response rate. However, the involvement of more younger age groups has been observed in other endemic sites [26]. Some studies have shown that, even amongst people with no detectable cell-mediated immunity to* Leishmania donovani*, the seroconversion rate decreased and the seroreversion rate increased with age [28].

More females demonstrated SCR as compared to males, though there is constant and clear male preponderance for CL in Sri Lanka [10, 15]. Male preponderance in disease prevalence is very likely to be the result of gender-dependent behavioural factors rather than true genetic factors. Occupational exposure (i.e., agricultural work, military activities in the rain forest) and social behavior patterns (i.e., outside during times of maximum vector activity) are known to increase the risk of CL as deciding factors for male preponderance [29]. Male preference in disease acquisition may depend on the behavioral factors in the study group.

Single lesions were associated with a higher SCR probably possibly due to the prominent host immune reactions when the lesion development and spread are minimal, resulting in more single lesions. However, different developmental stages were associated with a serological response. Small (≤2 cm) lesions have shown a higher serological response (46.7%). An association between the extent of immune reactions and lesion size has been shown before [30].

Antibody response was more evident in younger lesions than in older lesions. Time-dependent increase in host immune reactions with increased expression of IL-1 alpha, TNF-alpha, IL-10, and TGF-beta in lesions older than 4 months has been reported [31]. In general, serological response to LCL caused by* L. donovani *in Sri Lanka was seen in a wider patient population, though the numbers were inadequate to draw up valid conclusions based on the results.

## 5. Conclusions

First scientific evidence for a possible IgG antibody response associated with human cutaneous leishmaniasis in Sri Lanka is reported here. This may indicate presence of an immune response to a minor parasitemia in CL. However, ability of at least a proportion of CL infections to visceralize should not be disregarded. Visceral leishmaniasis has posed many difficulties in regional control initiatives in which Sri Lanka is not yet included [32, 33]. Limitations of the study to provide strong evidence for suggested possibilities include a relatively small sample size, failure to use an established serological tool for comparison, and lack of follow-up data.

This preliminary report highlights the need for more in-depth studies in a larger patient sample with improved investigation tools. Furthermore, there can be a relationship between the clinical outcome and the immunogenic properties of the parasites. Study group represented different characteristics; current findings can be considered as a projection on the locally acquired leishmanial infections in the island.

## Figures and Tables

**Table 1:** Comparison of ELISA results with LM.

	LM positive	LM negative	Total
ELISA			
Seropositive	14	3	17
Seronegative	21	12	33
Total	35	15	50

**Table 2:** Serological response according to different clinical features.

Clinical feature and category	Seropositive patients (*n*) (% within category)	Seronegative patients (*n*) (% within category)	Total (*n*) (% within category)
*Originating province*			
Northern	6 (42.9%)	8 (57.1%)	14 (100%)
Southern	9 (36.0%)	16 (64.0%)	25 (100%)
Other	2 (18.2%)	9 (81.8%)	11 (100%)
Total	17 (34.0%)	33 (66.0%)	50 (100%)
*Occupation* ^**∗**^			
Military	6 (30.0%)	14 (70.0%)	20 (100%)
Civilian	11 (39.3%)	17 (60.7%)	28 (100%)
Total	17 (35.4%)	31 (64.6%)	48 (100%)
*Age*			
Up to 20 years	3 (37.5%)	5 (62.5%)	8 (100%)
21–40 years	9 (39.1%)	14 (60.9%)	23 (100%)
>41 years	5 (26.3%)	14 (73.7%)	19 (100%)
Total	17 (34.0%)	33 (66.0%)	50 (100%)
*Sex*			
Male	13 (32.5%)	27 (67.5%)	40 (100%)
Female	4 (40.0%)	6 (60.0%)	10 (100%)
Total	17 (34.0%)	33 (66.0%)	50 (100%)
*Number of lesions*			
Single	14 (37.8%)	23 (62.2%)	37 (100%)
Multiple	3 (23.1%)	10 (76.9%)	13 (100%)
Total	17 (34.0%)	33 (66.0%)	50 (100%)
*Size of the lesions*			
Up to 2 cm	14 (46.7%)	25 (53.3%)	39 (100%)
>2 cm	3 (27.2%)	8 (72.8%)	11 (100%)
Total	17 (34.0%)	33 (66.0%)	50 (100%)
*Duration*			
Up to 3 months	4 (30.8%)	9 (69.2%)	13 (100%)
3–6 months	7 (36.8%)	12 (63.2%)	19 (100%)
7–9 months	2 (28.6%)	5 (71.4%)	7 (100%)
10–12 months	4 (36.4%)	7 (63.6%)	11 (100%)
Total	17 (34.0%)	33 (66.0%)	50 (100%)
*Type of lesion*			
Papule	1 (25.0%)	3 (75.0%)	4 (100%)
Nodule	8 (47.1%)	9 (52.9%)	17 (100%)
Ulcerating nodule	4 (30.8%)	9 (69.2%)	13 (100%)
Ulcer	3 (30.0%)	7 (70.0%)	10 (100%)
Plaque	1 (16.7%)	5 (83.3%)	11 (100%)
Total	17 (34.0%)	33 (66.0%)	50 (100%)

^*∗*^Missing cases excluded.
